# Haemodynamic Effects of Lung Recruitment Manoeuvres

**DOI:** 10.1155/2015/478970

**Published:** 2015-11-22

**Authors:** András Lovas, Tamás Szakmány

**Affiliations:** ^1^Department of Anaesthesiology and Intensive Therapy, University of Szeged, Semmelweis Utca 6, Szeged 6725, Hungary; ^2^Department of Anaesthesia, Intensive Care and Pain Medicine, Cardiff University, Institute of Infection and Immunology, Heath Park Campus, Cardiff CF14 4XN, UK; ^3^Aneurin Bevan University Health Board, Critical Care Directorate, Royal Gwent Hospital, Cardiff Road, Newport NP20 5UB, UK

## Abstract

Atelectasis caused by lung injury leads to increased intrapulmonary shunt, venous admixture, and hypoxaemia. Lung recruitment manoeuvres aim to quickly reverse this scenario by applying increased airway pressures for a short period of time which meant to open the collapsed alveoli. Although the procedure can improve oxygenation, but due to the heart-lung and right and left ventricle interactions elevated intrathoracic pressures can inflict serious effects on the cardiovascular system. The purpose of this paper is to give an overview on the pathophysiological background of the heart-lung interactions and the best way to monitor these changes during lung recruitment.

## 1. Introduction

Patients admitted to the intensive care unit are often affected by acute respiratory distress syndrome (ARDS). ARDS is a life-threatening condition precipitated by disorders frequently resulting in critical care admissions like trauma, severe burns, sepsis, pancreatitis, and pneumonia [1]. All of these disorders, either causing direct (pulmonary) or indirect (extrapulmonary) tissue damage, are featured by a systemic inflammatory response. The released cytokines like interleukin- (IL-) 1, IL-6, IL-8, and tumor necrosis factor activate neutrophils in the lung throughout the inflammatory cascade [2]. The activated immune cells excrete injurious substances such as free oxygen radicals and proteolytic enzymes leading to alveolar endothelium and epithelium destruction. The latter pathophysiological mechanism induces impaired permeability in the lung resulting in alveolar immerging by the protein-rich oedema fluid [3]. Surfactant, which has a major role in modulating the surface tension of alveoli, is also washed out. Furthermore, the surfactant production is also decreased due to the dysfunction of type II epithelial cells. As a consequence, pulmonary atelectasis develops due to alveolar collapse [4].

Pulmonary atelectasis is accompanied by arterial hypoxaemia due to increased intrapulmonary shunt [5]. As severe acute hypoxaemia is a potential danger for all vital organs, its resolution is of pivotal importance. There are several interventions, which may help improve oxygenation. In the most severe circumstances, extracorporeal membrane oxygenation [6], high frequency oscillatory ventilation [7], and prone positioning [8] have been shown to reverse persistent hypoxaemia. Some of these interventions require special equipment, demand extra manpower, and may be time consuming to commence. In less severe cases of acute hypoxaemia, especially when this is primarily caused by atelectasis, the collapsed lung areas can be opened up with the help of transient increment in transpulmonary pressure (TP) within a short time, hence decreasing shunt fraction and improving arterial oxygenation [9]. This procedure is called the lung recruitment manoeuvre. It can be accompanied by the titration of the “optimal” PEEP, a process which is called on a broader term the “open lung concept” described by Lachmann in 1992 [10].

Several applications of recruitment manoeuvres have been described so far. Although these may differ in certain details but by-and-large the most common feature is in all of them that they apply peak airway pressures of 40–60 cm H_2_O for a short period of time, usually not exceeding 40–120 seconds [9]. Although survival benefit has not been demonstrated with any of the recruitment manoeuvres, the intervention is frequently administered in atelectasis induced hypoxaemia [11]. It is beyond the scope of this paper to review the available recruitment techniques; therefore we will only concentrate on the heart-lung interactions, haemodynamic effects, and the monitoring alternatives.

The anatomical proximity of the lungs and heart within the chest means that transiently increased intrathoracic pressures have major effect on systemic cardiovascular function. Undesired side effects of the recruitment process mainly arise from the increased airway pressures which can cause overdistension of alveoli in well-ventilated lung areas, marked increase in ventilation-perfusion mismatch, barotrauma, pneumothorax, and new air leak around an existing chest tube [12]. These effects may be even more pronounced in patients with ARDS in whom haemodynamic instability is a common feature [13]. It has strong pathophysiological rationale supported by clinical data that routine ICU monitoring, such as invasive blood pressure and central venous pressure monitoring, may not be adequate to follow haemodynamic changes encountered during lung recruitment [14].

## 2. Effects on Right Heart and Pulmonary Circulation

Distending lung volume evoked by applied raised airway pressure leads to an increase in TP. TP can be estimated from the difference between alveolar and intrathoracic pressures. The transmission of TP to the pleural space impedes venous return and the filling of the right ventricle. Meanwhile, the increased TP is transposed to vessels interlacing the lung tissue hereby elevating pulmonary vascular resistance (PVR) and right ventricular afterload.

### 2.1. Systemic Venous Return and Right Ventricle Preload

The increase of intrathoracic pressure compresses the right atrium and caval veins carrying the systemic venous return to the heart. The generated retrograde pressure results in elevation in the central venous pressure (CVP) and could impede right ventricular filling. Due to this mechanism the assessment of cardiac preload by CVP during lung recruitment manoeuvre is misleading, as the transmission of the intrathoracic pressure to the intravascular compartment [14] does not represent the true preload component. Restricted right ventricular preload is a dominant but not solitary mechanism in the fall of the right ventricular ejection fraction.

### 2.2. Right Ventricular Afterload

Right ventricular afterload represents the resistance, quantified by the pulmonary artery pressure, which the right ventricle should overcome to eject the blood through the pulmonary valve. During the lung recruitment manoeuvre, the interposed TP further increases the area where the intraluminal pressure of the juxta-alveolar capillaries is lower than the intra-alveolar pressure. This results in a significant increase in the pulmonary vascular resistance, parallel to an increase in the pulmonary artery pressure. Thus right ventricular afterload is augmented by transitionally elevated TP.

Hypoxic pulmonary vasoconstriction, which developed to attenuate the ventilation-perfusion mismatch caused by alveolar hypoventilation, is another important determinant of the right ventricular afterload [15]. During significant hypoxaemia, the atelectatic lung regions are served with only marginal circulation. The hypoxic pulmonary vasoconstriction contributes to the overall pulmonary vascular resistance; however there is only limited data about its role and the changes occurring during lung recruitment.

**Figure 1:**
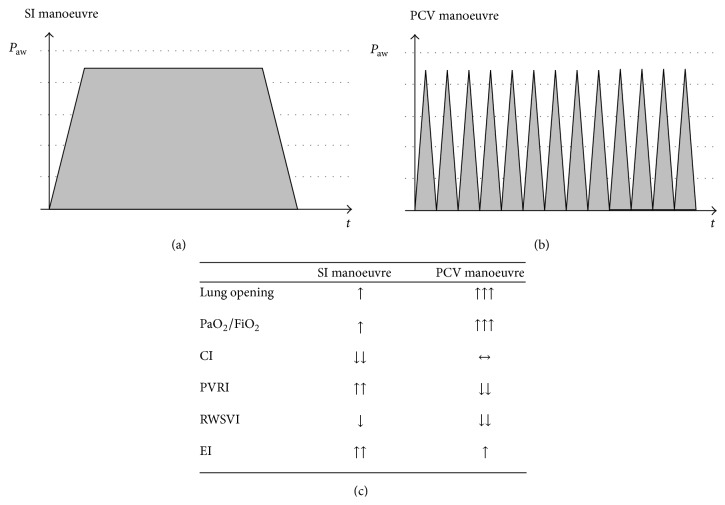
Pressure-time product (a-b) and main characteristics (c) of sustained inflation (SI) and pressure control ventilation (PCV) recruitment manoeuvres. *P*_aw_, airway pressure; *t*, time; CI, cardiac index; PVRI, pulmonary vascular resistance index; RWSVI, right ventricle stroke work index; EI, eccentricity index.

These mechanisms can impair right ventricular function and decrease right ventricular stroke volume. Iannuzzi et al. [16] found that pressure controlled ventilation (PCV) with peak inspiratory pressure of 45 cm H_2_O for 2 minutes generated a higher grade of lung opening and resulted in a major and significant increase in PaO_2_/FiO_2_ ratio compared to sustained inflation (SI) (Figure 1). They found that hypoxic pulmonary vasoconstriction and pulmonary vascular resistance index (PVRI) were also reduced, with smaller degree of hyperinflation in the PCV group (Figure 1).

On the other hand, Reis Miranda et al. could not detect any significant impairment in right heart function comparing conventional mechanical ventilation to the open lung concept in patients after cardiac surgery [17]. This disagreement between the two observations may arise from the different methods applied, especially the different timing of measurements. Whereas Iannuzzi et al. investigated the immediate effects [16], in the other study cardiovascular measurements were taken every 30 minutes for 3 hours [17].

Similar results were reported by Celebi et al. [18] where pulmonary and haemodynamic effects of two different recruitment manoeuvres were investigated in patients after open heart surgery. During the study period, there was no significant change of PVR between the groups, measured after 15 minutes. One may suggest from these observations that the effect of the recruitment manoeuvre on the right ventricular afterload is transient, lasting for seconds only. Relieving high airway pressures after recruitment helps in the normalization of haemodynamic changes in the pulmonary circulation.

Apart from the different methods employed in the studies, controversial results may arise from the different patient inclusion criteria. In the study of Iannuzzi et al., patients with primary ARDS due to hospital acquired bacterial pneumonia were recruited [16], whilst Reis Miranda et al. investigated patients following cardiac surgery without significant lung injury [17]. It has been demonstrated in a murine model of acute lung injury induced by* Escherichia coli* lipopolysaccharide that dynamic inflation applied during lung recruitment produced increased right ventricular pressure and total PVR. It also resulted in sustained inflammation and vascular dysfunction whilst no similar changes were reported in healthy lungs [19].

### 2.3. Right Ventricular Ejection Fraction

**Figure 2:**
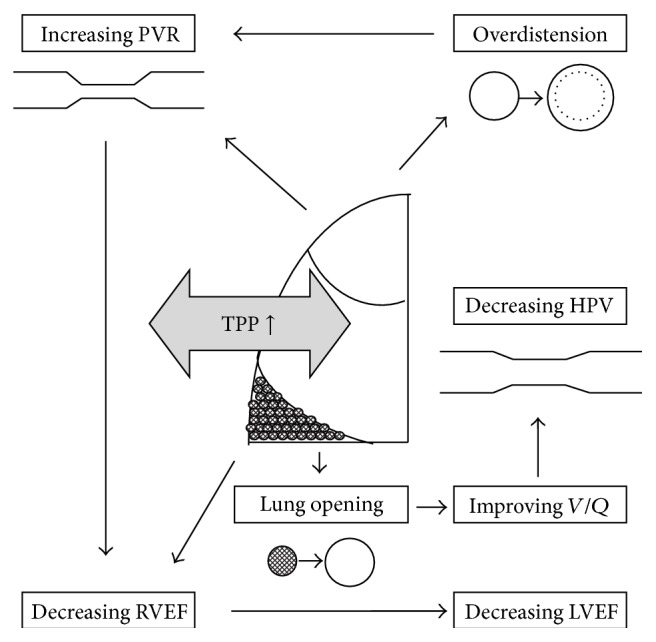
The effects of increased transpulmonary pressure (TPP). PVR, pulmonary vascular resistance; RVEF, right ventricular ejection fraction; LVEF, left ventricular ejection fraction; *V*/*Q*, ventilation/perfusion; HPV, hypoxic pulmonary vasoconstriction.

**Figure 3:**
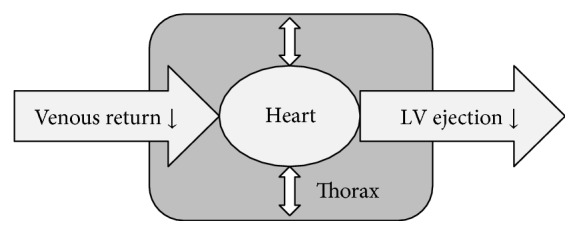
Pressure chamber (heart) in a pressure chamber (thorax). LV ejection; left ventricular ejection.

The primary role of the right ventricle is to receive systemic venous blood and to forward it via a high volume and low-pressure system, the pulmonary circulation, to the left heart chambers [20]. Right ventricular ejection fraction is affected by preload, contractility, and afterload. During the recruitment manoeuvre, the raised intrathoracic and right atrial pressures, as discussed previously, could affect both venous return and afterload significantly [18] (Figures 2 and 3). Both mechanisms can result in impaired right ventricular contractility.

Right ventricular ejection fraction is inversely related to the ventricle's afterload. In the study by Reis Miranda et al. [17], PVRI and right ventricular ejection fraction showed no significant changes following recruitment within the first 3 hours, neither within nor between groups at any measurement points. However, if immediate effects on right ventricular function were investigated, then significant increase in right ventricular stroke work index was reported during the recruitment manoeuvre and 2 minutes following the intervention [21]. These results call for further attention to the immediate effects of the recruitment manoeuvre on right heart function. There is also lack of data, whether it has any clinically relevant long-term effects.

### 2.4. Ventricular Interdependence

**Figure 4:**
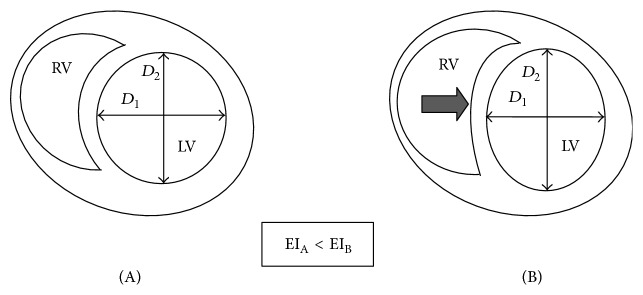
Ventricular interdependence before (A) and during alveolar recruitment manoeuvre (B). *D*_1_, midmitral diameter; *D*_2_, diameter orthogonal to *D*_1_. Eccentricity index (EI) is calculated as *D*_2_/*D*_1_. RV, right ventricle; LV, left ventricle.

It is important to note that the end-diastolic right ventricular volume has a direct effect on the left ventricle, which holds true* vice versa*. This is called the ventricular diastolic interdependence [22]. The two chambers are coupled within a common pericardial sac and share joint intraventricular septa as a traverse wall. Thus, their volumes are limited by the pericardium; hence any change in the right ventricular end-diastolic volume has an effect on the left ventricular end-diastolic volume (Figure 4).

During sigh recruitment, the right ventricle can have a marked effect on the adjacent heart chamber. When lung recruitment manoeuvre is applied by a sustained inspiration, left ventricular end-diastolic area can be reduced by as much as 45% [23]. PVR is also increasing with the transposed intrathoracic pressure, leading to an acute right ventricular pressure overload with dilation, leftward septal shift, and left ventricular collapse resulting in low cardiac output (CO) and marked systemic haemodynamic changes (Figure 4). These changes are transient and only seen during the manoeuvre, with almost instant normalisation of haemodynamics once the intrathoracic pressure returns to the baseline [16].

There is a special scenario when this interdependence is questioned and this is the postoperative period after cardiac surgery when the pericardial sac is kept open [17]. Theoretically, due to the missing pericardial sac, interaction between the two adjacent ventricles should be impaired and in these patients the diastolic interdependence is not fully present. However, in an animal experiment on dogs, it was found that artificially increasing the pulmonary artery resistance and the right ventricular load had a profound effect on the left ventricular filling dynamics. This was explained by the prolonged relaxation and altered pressure-volume chamber relations [24].

## 3. Effects on Left Heart and Systemic Circulation

The cardiopulmonary system is described by Pinsky as a pressure chamber inside a pressure chamber [25]. Any increment in the intrathoracic pressure increases the right atrial pressure and decreases the venous return and the transmural left ventricular systolic pressure, hence attenuating the left ventricular ejection fraction (Figure 3). If haemodynamic changes are solely monitored by mean arterial pressure (MAP) during lung recruitment manoeuvre, one can theoretically miss relevant alterations in the systemic circulation. Recent investigations concluded that simple haemodynamic parameters like MAP or heart rate did not show any significant change during and after various recruitment interventions [17, 21, 26]. However, applying advanced invasive haemodynamic monitoring, relevant changes in the systemic circulation can be observed [14].

### 3.1. Left Ventricle Preload and Afterload

As described above, ventricular interdependence plays a significant role during lung recruitment manoeuvre in determining the left ventricular preload. The increased TP compresses the right atrium and increases the CVP by the transmission of pressure to the intraluminal compartment of the caval veins. Echocardiographic investigations identify this mechanism as partial cause of the impaired left ventricular preload and consecutive decrease of CO [16].

Left ventricular afterload is defined as the pressure of the wall in the left ventricle during ejection. Following Laplace's law, if there is no significant alteration in the systolic arterial pressure, as seen throughout most of the studies investigating recruitment manoeuvre, left ventricle afterload decreases along with the fall of the transmural pressure of the left ventricle [27]. Measuring these pressure fluctuations requires sophisticated methods at the bedside; therefore correlation between left ventricle afterload and lung recruitment has not been investigated thoroughly in human subjects.

### 3.2. Cardiac Output and Left Ventricular End-Diastolic Volume

The increased availability of sophisticated continuous CO monitoring using pulse pressure analysis like pulse contour cardiac output (PiCCO), lithium dilution cardiac output (LiDCO), or FloTrac/Vigileo techniques and Doppler cardiac output devices enabled the clinicians to follow alterations in the systemic haemodynamics during each cardiac cycle [28]. Utilising these advanced monitoring techniques, profound and significant decrease in CO was observed during lung recruitment manoeuvres [14, 16, 23]. This decline in left ventricular performance can be explained by interconnected fluctuations within the “chamber in the chamber” system discussed previously [25]. Increased intrathoracic pressure, decreased right ventricular filling, and increased right ventricular outflow impedance with leftward intraventricular septal shift are all precipitating reduced CO (Figure 2). However, rapid recovery of the baseline CO was described when the effects were measured in a temporal study, so the depression is only transient correlating with the temporarily increased TP [29].

The absolute reduction in CO is influenced by the technique of the lung recruitment and also by the nature of the lung injury. As discussed previously, sustained inflation manoeuvre can significantly change left ventricle eccentricity index (Figure 4) indicating a significant reduction in left ventricular end-diastolic volume compared to PCV-recruitment, which was accompanied by a less profound effect.

The importance of the underlying pathology of the lung injury has been emphasised by Lim et al. [29]. They investigated three different types of lung injury models during recruitment: oleic acid injury depicting acute surfactant loss, ventilator-induced lung injury, and finally an injury caused by infection. Animals in all three models underwent a PCV, a sigh, and a PEEP incremental recruitment. Regardless of the way the manoeuvres were executed, a significant but interim drop of CO was observed in each model. However, in the pneumonia model, the CO decreased to a greater extent and the recovery of systemic haemodynamics also showed a moderate pattern as compared to the other two. It is possible that, in septic shock induced inflammatory response, a more profound depression of myocardial function and compensatory vasomotor reflexes takes place [30]. Out of the three recruitment techniques, the sigh manoeuvre resulted in the most significant reduction in CO in accordance with previous investigations [16, 29].

One of the available methods to prevent the undesired decrease in CO during lung recruitment is the selective lung opening technique described by Hansen et al. in an elegant animal model [31]. In their experiment, pigs were randomized into two groups of lung recruitment manoeuvres (by applying 40 cm H_2_O airway pressure for 30 seconds), either a selective lung recruitment manoeuvre such as using the inner lumen of the bronchial blocker followed by a whole lung recruitment manoeuvre or* vice versa*. Whilst there was no significant difference in the improvement of oxygenation and the end-expiratory lung volume between the two groups, there were no circulatory changes during the selective technique. On the other hand, the whole lung recruitment caused a significant drop in CO and left ventricular end-diastolic volume. This suggests that selective lung recruitment technique might be advantageous in patients with lobar atelectasis prone to haemodynamic instability. However, this new method requires further investigations in humans.

### 3.3. Alterations in Heart Rate

Along with stroke volume, heart rate is the other determinant of CO. Through the recruitment manoeuvre, one may expect the development of reflex tachycardia along the drop in CO. Many investigations failed to observe such an increase in heart rate; principally no significant alteration of pulse rate was found [14, 16–18, 21, 26]. However, in the investigation of Nielsen et al., the significant reduction in heart rate was suspected as the major component of the declining CO during the sigh manoeuvre [23]. One of the explanations is that the inflated lung tissue can activate vagal tone causing bradycardia [32]. Another assumption is that the sigh manoeuvre may precipitate a similar pattern in intrathoracic pressure as the Valsalva manoeuvre, hence producing reduction in heart rate. As opposed to the previous findings, Lim et al. reported an increased heart rate, perhaps reflecting just a sympathetic response to the lengthy recruitment procedure they used [33].

### 3.4. The Effect of Volemic State on Left Heart Function

One of the main patient exclusion criteria in the lung recruitment studies is haemodynamic instability and/or signs of intravascular volume depletion [14, 18, 23, 26, 33]. Hypovolaemia can amplify the undesirable haemodynamic effects of lung recruitment manoeuvre as demonstrated by Nielsen et al. [34]. In their animal experiment, the impact of recruitment manoeuvre on central haemodynamics was investigated in pigs with different volemic states. The animals were randomized to a 10-second-long recruitment followed by lung opening lasting 30 seconds by applying 40 cm H_2_O airway pressure or* vice versa*, performed under hypo-, normo-, and hypervolemia. Volemic states were controlled either by removing 15% of the estimated blood volume or by infusion of a volume equal to 15% of the estimated blood volume with 3% dextran in Ringer's solution. The study focused on the immediate circulatory effects. They found a significant reduction in left ventricular end-diastolic volume, which could explain the depleted CO during lung recruitment manoeuvre in pigs with acute lung injury. As expected, the impact of this effect was significantly exaggerated by hypovolaemia. On the other hand, hypervolemic conditions prevented the reduction of CO during the extended sigh manoeuvre.

Fougères et al. suggested that some microvessels of the lungs may be collapsed by PEEP and were recruitable by the increased left ventricular preload [35]. In their recent investigation in patients with ARDS, recruitment was accomplished by increasing PEEP for reaching a plateau pressure of 30 cm H_2_O. During the manoeuvre, CO was decreased along with increasing right ventricular afterload. Importantly, passive leg raising restored the CO and reduced the PVR. These important observations reinforce the need of appropriate intravascular volume assessment prior to the alveolar opening procedure.

## 4. Recruitment in Spontaneously Breathing Patients

There is some evidence that patients on continuous positive airway pressure and pressure support ventilation may benefit from recruitment manoeuvres, resulting in fast and significant improvement in oxygenation [26]. However, there are very few publications in this topic as most studies on recruitment were performed in patients receiving controlled mechanical ventilation. Although it is well known that spontaneous ventilation and spontaneous breathing efforts significantly interfere with heart-lung interactions, apart from routine parameters such as blood pressure and heart rate, we have no detailed haemodynamic information in this patient group. As the changes are markedly different from that observed during controlled ventilation, this can be a potential field for further research.

## 5. Conclusions

Applying recruitment technique is a simple procedure to perform at the bedside but it is not free of certain risks. Increased airway and intrathoracic pressures can inflict deleterious haemodynamic effects due to the anatomical proximity of the lungs and heart within the thoracic cavity. Therefore, detailed understanding of the physiology and pathophysiology of these changes is necessary to perform lung recruitment safely. The evidence suggests that those patients who are at risk of overt hypovolaemia or whose lung injury is secondary to a primary lung infection, hence developing significant localised inflammatory changes, are more likely to benefit from advanced haemodynamic monitoring by devices that enable continuous and reliable evaluation of cardiac output during lung recruitment so that the treating clinician can maintain circulatory homeostasis.
